# Supplementary material for: MetaRibo-Seq measures translation in microbiomes
Source: Nat Commun. 2020 Jun 29;11:3268. doi: 10.1038/s41467-020-17081-z (PMC7324362; doi:10.1038/s41467-020-17081-z)
Supplement: Supplementary file 10 — Supplementary Data 7 [file 41467_2020_17081_MOESM10_ESM.zip › File2/Confidence_High_Taxonomy/184118_out.krona.html]

Javascript must be enabled to view this page.

members
magnitude
magnitudeUnassigned
count
unassigned
taxon
rank

184118\_out

4

2
superkingdom
4

phylum
4
976

200643
4
class

4
order
171549

4
family
815

816
genus
4

329854

SRS018623\_contig\_number\_19276SRS019808\_contig\_number\_contig-100\_4594.85054SRS076756\_contig\_number\_17120SRS144362\_contig\_number\_3145
4
species
